# Revolving ATPase motors as asymmetrical hexamers in translocating lengthy dsDNA via conformational changes and electrostatic interactions in phi29, T7, herpesvirus, mimivirus, *E. coli*, and *Streptomyces*

**DOI:** 10.1002/EXP.20210056

**Published:** 2023-02-05

**Authors:** Tao Weitao, Giovanna Grandinetti, Peixuan Guo

**Affiliations:** ^1^ UT Southwestern Medical Center Center for the Genetics of Host Defense Dallas TX USA; ^2^ College of Science and Mathematics Southwest Baptist University Bolivar MO USA; ^3^ Center for Electron Microscopy and Analysis The Ohio State University Columbus OH USA; ^4^ Center for RNA Nanobiotechnology and Nanomedicine Division of Pharmaceutics and Pharmacology, College of Pharmacy Dorothy M. Davis Heart and Lung Research Institute, James Comprehensive Cancer Center, College of Medicine The Ohio State University Columbus OH USA

**Keywords:** ATPase, bacteriophage phi29, DNA packaging, DNA translocase, hexamer, Viral DNA packaging motor

## Abstract

Investigations of the parallel architectures of biomotors in both prokaryotic and eukaryotic systems suggest a similar revolving mechanism in the use of ATP to drive translocation of the lengthy double‐stranded (ds)DNA genomes. This mechanism is exemplified by the dsDNA packaging motor of bacteriophage phi29 that operates through revolving but not rotating dsDNA to “Push through a one‐way valve”. This unique and novel revolving mechanism discovered in phi29 DNA packaging motor was recently reported in other systems including the dsDNA packaging motor of herpesvirus, the dsDNA ejecting motor of bacteriophage T7, the plasmid conjugation machine TraB in *Streptomyces*, the dsDNA translocase FtsK of gram‐negative bacteria, and the genome‐packaging motor in mimivirus. These motors exhibit an asymmetrical hexameric structure for transporting the genome via an inch‐worm sequential action. This review intends to delineate the revolving mechanism from a perspective of conformational changes and electrostatic interactions. In phi29, the positively charged residues Arg‐Lys‐Arg in the N‐terminus of the connector bind the negatively charged interlocking domain of pRNA. ATP binding to an ATPase subunit induces the closed conformation of the ATPase. The ATPase associates with an adjacent subunit to form a dimer facilitated by the positively charged arginine finger. The ATP‐binding induces a positive charging on its DNA binding surface via an allostery mechanism and thus the higher affinity for the negatively charged dsDNA. ATP hydrolysis induces an expanded conformation of the ATPase with a lower affinity for dsDNA due to the change of the surface charge, but the (ADP+Pi)‐bound subunit in the dimer undergoes a conformational change that repels dsDNA. The positively charged lysine rings of the connector attract dsDNA stepwise and periodically to keep its revolving motion along the channel wall, thus maintaining the one‐way translocation of dsDNA without reversal and sliding out. The finding of the presence of the asymmetrical hexameric architectures of many ATPases that use the revolving mechanism may provide insights into the understanding of translocation of the gigantic genomes including chromosomes in complicated systems without coiling and tangling to speed up dsDNA translocation and save energy.

## Graphical abstract

The revolving DNA motors of ATPase have been found in viruses (phi29, T7, herpesvirus, and mimivirus) and bacteria (*E. coli* and *Streptomyces*). The motors made of asymmetrical 6 subunits translocate lengthy double‐stranded (ds)DNA. The dsDNA is moved in a circular motion around the inner surface of the motor channel subunits. The translocation involves conformational changes and electrostatic interactions between the motor subunits and DNA.

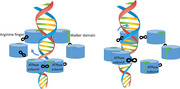

## 1 INTRODUCTION

DNA is the blueprint for life, containing instructions for the generation of all of the proteins that make up different organisms. These include the proteins necessary for manipulating nucleic acids in such processes as repair, recombination, transcription, and translation. Some of the ATPase proteins that directly interact with DNA contain a hexameric ring that encloses DNA and translocates it. The proteins of this type are generally termed DNA motors. These biomotors are of special interest because their efficacy in transporting large biomolecules can be applied for therapeutic and industrial use. Additionally, these hexameric motors may be valuable drug targets, since their high stoichiometry and dependence on sequential mechanisms for transport make them easier targets than monomeric proteins.^[^ ^1^ ^]^

Transporting long strands of DNA is no easy task. In order to overcome the energetic barriers of DNA transportation, ATP is used. However, the exact mechanisms for how these motors worked remained elusive for many years. Initially, it was thought that there were two mechanisms for moving DNA, the linear and the rotational mechanism. For the linear mechanism, a DNA‐binding protein “walks” along a DNA strand, while in the rotational mechanism, the hexameric ring acts as a nut and the DNA acts as a bolt.^[^ ^2^ ^]^ More recently, a third mechanism was revealed, in which DNA travels around the inner channel of the hexameric ring, and translocation through the ring is driven by conformational changes of the ring proteins without the need for the DNA or the channel to rotate.^[^ ^3^, ^4^, ^5^ ^]^ Such an action offers several advantages to the revolution mechanism compared with the rotation mechanism, including avoiding supercoiling that requires the work of topoisomerase enzymes to correct.^[^ ^6^, ^7^ ^]^

The double‐strand (ds)DNA‐packaging motors of prokaryotic cells and their viruses that pack the genomes against an accumulating internal pressure in an entropically unfavorable manner have evolved common structures and mechanisms.^[^ ^8^, ^9^, ^10^, ^11^ ^]^ Among these, the motor of bacteriophage phi29 was the first to be constructed as a defined in vitro system.^[^ ^12^ ^]^ The packaging of the phi29 DNA genome and the production of the infectious virion through this in vitro system provide the initial evidence for the hypothesis stating that viral DNA packaging does not rely on host proteins and enzymes. The study about the function of the viral DNA packaging protein gp16 in 1986^[^ ^13^ ^]^ demonstrated the following key features about the DNA packaging enzymes of the bacteriophage. First, the DNA packaging enzymes were discovered to be ATPases (Figure 6A). Second, the characterization of ATPases from multiple systems led to the identification of the conserved sequence of GxxGxGKT/S, which was termed the Walker A sequence. The Walker A motif was found to be highly conserved across the domains of life and their viruses, and was eventually awarded a Nobel Prize in 1997.^[^ ^14^ ^]^ Third, one ATP molecule was spent on the packaging of 2 DNA base pairs, close to the current calculation of 1.75 base pairs per ATP. Fourth, gp16 was found to be a DNA‐dependent ATPase. Fifth, it has been demonstrated that gp16 was bound to DNA and changed its conformation upon ATP hydrolysis. Sixth, the DNA packaging motor was made of one pair of proteins: an ATPase and a DNA binding protein. These are the features of the phage motor that have been found common to the revolving motors of diverse systems.

The revolving motion of DNA translocation was also originally discovered in the phi29 motor.^[^ ^3^, ^15^ ^]^ Several clues pointed to the existence of the revolving mechanism rather than the rotational “nut and bolt” mechanism. For a nut and bolt to be effective, the nut must fit tightly around the bolt. This was not the case in the phi29 motor. The diameter of the motor channel is almost twice as large as the diameter of dsDNA.^[^ ^16^ ^]^ Additionally, for a nut and bolt to be functioning, they have to be threaded the same way. The phi29 motor channel displays left‐handed chirality, opposite of the right‐handed chirality of dsDNA.^[^ ^16^ ^]^ Lastly, for the nut and bolt mechanism to hold true, either the nut or the bolt has to rotate. Low‐resolution Cryo‐EM images showed dsDNA bending around the portal region,^[^ ^17^, ^18^ ^]^ and a tilting of dsDNA when passing through the connector channel.^[^ ^19^ ^]^ Further evidence defining the revolving mechanism came from the following functional analyses. First, when the T4 motor active in dsDNA packaging was fused to the procapsid protein, thus rendering it incapable of rotating, the motor was still able to translocate dsDNA.^[^ ^20^ ^]^ Second, single‐molecule force spectroscopy combined with polarization spectroscopy studies did not show any evidence of the motor or DNA rotating.^[^ ^21^ ^]^ Third, bead clusters attached to both ends of the DNA substrate showed no sign of complete rotation during the active packaging of dsDNA,^[^ ^22^ ^]^ and the dsDNA substrate twists about 1.5°/base pair (bp) instead of a full 360°/complete helical turn.^[^ ^23^ ^]^ In fact, this finding verifies a revolving instead of a rotational mechanism because if one helical turn of dsDNA is made of ∼10.5 bases, then 1.5° /bp × 10.5 bp/turn = 15.7°, which is far smaller than 360°. The data from several studies indeed demonstrate a revolving mechanism by which the dsDNA substrate revolves forward through the motor channel. Fourth, translocation of the dsDNA substrate is one‐way traffic coordinated by the various motor components. The directional translocation is maintained by a ratchet‐like mechanism making use of multiple functional features of a channel, ATP‐coupled conformational alternations, and the recognition of the 5′‐3′ strand for single‐directional movement. Moreover, this revolving mechanism of translocation is executed by biomotors in a great variety of biological systems, such as bacteria and the dsDNA viruses (bacteriophages) as well as eukaryotic dsDNA viruses (Table 1).^[^ ^8^, ^9^, ^10^, ^11^, ^24^, ^25^ ^]^

**TABLE 1:** Common characteristics of the hexameric DNA packaging motors

DNA translocation motors	Organism of biological systems	Components	Structure	Motor coordination	Translocation mechanism
gp16	Phi29 of bacteriophages	Hexameric gp16 ATPase, hexameric pRNA and dodecameric connector	Co‐axial hexameric rings formed gp16, pRNA, and connector	Transient dimerization via the arginine finger	Phage genome packaging by the revolving push through one‐way valve mechanism
gp15	T7 of bacteriophages	Core proteins gp15 ATPase and gp16	Hexameric rings of gp15 and gp16	The energy coupling by the arginine finger	Phage genome packaging by the revolving mode
pUL15	Herpesvirus of eukaryotic viruses	Heterotrimers of pUL15 ATPase, pUL28, and pUL33	Hexameric rings of pUL12, pUL28 and pUL33	The role of the arginine finger is not fully defined	Viral genome packaging by the revolving motion
TraB	Plasmid of bacteria	Membrane‐associated DNA‐dependent ATPase	Hexameric ring of TraB	Unknown	Bacterial plasmid conjugation by the revolving mechanism
FtsK	Bacteria of prokaryotes	Membrane‐associated DNA‐dependent ATPase containing α, β, and γ domains; α and β have ATPase activity	Hexameric rings of α and β	Unknown	Resolving the chromosomal dimers by a α‐β hinged movement
APMV packaging motor	Mimivirus of eukaryotic viruses	FtsK‐related	FtsK‐related	Unknown	Putative viral genome resolution and packaging

These revolving biomotors of the prokaryotic and eukaryotic systems have the following basic structures and functions in common (Table 1). They possess a hexameric ATPase ring connected to a central channel, and dsDNA is translocated through both of them. Among the viruses and plasmids of the prokaryotic system, the dsDNA bacteriophages such as phi29 and T4 have motors that play a dual role in injection of the viral genomes into the bacterial hosts and in the translocation of the duplicated viral genomes into the procapsids during the phage assembly. A plasmid‐encoded protein TraB of the soil bacterial genus *Streptomyces* transfers plasmid DNA in a unique process. Similarities in sequence and structure as well as DNA binding characteristics of TraB to those of FtsK (Filamenting temperature‐sensitive mutant K) demonstrate that TraB is an FtsK‐like ancestor protein, suggesting the adaptation relationship between FtsK/SpoIIIE and TraB.^[^ ^26^ ^]^ For the bacterial cellular motor, FtsK that comprises a family of motor proteins coordinates the replication and segregation of the chromosome during cell division.^[^ ^27^ ^]^ FtsK helps resolve bacterial chromosome dimers during cell division by translocating dsDNA in a mechanism similar to filament treadmilling.^[^ ^28^ ^]^ Of the viruses in the eukaryotic system, herpesvirus possesses a genome packaging machinery in which terminase complex displays a hexameric ring structure, an architecture suggesting a sequential revolving motion for DNA translocation.^[^ ^29^ ^]^ The dsDNA packaging motors of mimivirus exist in the mimiviridae, a highly diverse family of dsDNA viruses^[^ ^30^, ^31^ ^]^ infecting many aquatic eukaryotes.^[^ ^32^ ^]^ They are evolutionarily related to FtsK (Table 1).^[^ ^33^ ^]^ While a wealth of structural and functional evidence supports the revolving mechanism for dsDNA translocation, our understanding remains fragmented about the interplay among force generation, conformational changes, and electrostatic interactions of the motor components. The mechanism is not fully explored in the eukaryotic system. In this review, we present the interactions of the phi29 motor components in the context of force generation for the hexametric ATPase activity by which DNA is translocated. This understanding may shed light on the related DNA motors of the bacterial cellular and eukaryotic counterparts.

## 2 PARALLEL ARCHITECTURES AND FUNCTIONS OF THE VIRAL AND CELLULAR MOTORS

### 2.1 “Push through one‐way valve” mechanism of a bacteriophage motor

**FIGURE 1:**
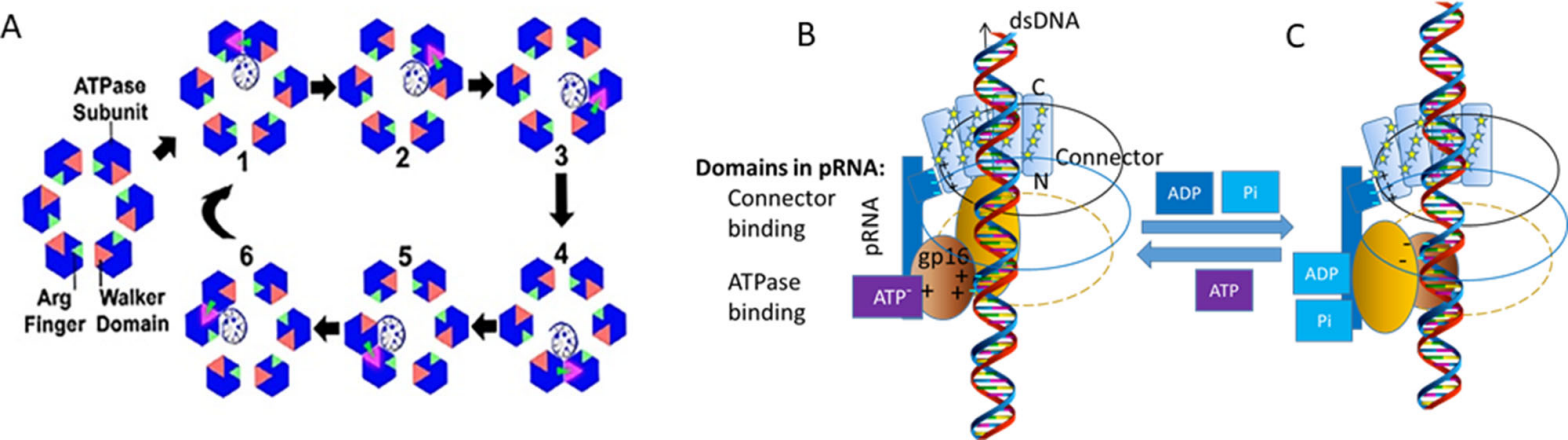
Models for the dsDNA translocation by the phi29 motor showing the interplay between conformational changes and electrostatic interactions in dsDNA translocation by the phi29 DNA packaging motor. (A) A top view of (B) and (C) to illustrate the dynamic revolving mechanism. Reproduced with permission.^[^ ^41^ ^]^ Copyright 2019, American Chemical Society. (B) The hexameric pRNA ring braces the hexameric gp16 ATPase and the dodecameric connector by electrostatic force. The three basic residues Arg‐Lys‐Arg in the N‐terminus of the connector are indicated as +, essential for binding to the − charged interlocking domain of pRNA by electrostatic force. ATP binding to an ATPase subunit via electrostatic force induces the closed conformation of ATPase. The ATP‐bound subunit associates with an adjacent subunit to form a dimer via the arginine finger as shown in (A). The ATP‐bound subunit in the dimer increases its affinity for the negatively charged dsDNA via dielectric allostery. (C) ATP hydrolysis induces the expanded conformation that lifts up DNA. The (ADP + Pi)‐bound subunit in the dimer of ATPase repels dsDNA. The + charged lysine rings of the connector attract the − charged dsDNA periodically to keep revolving motion of dsDNA along the channel while the gating property of the connector maintains the one‐way translocation of dsDNA

The phi29 motor encompasses one ring of the dodecameric gp10 connector, one ring of the hexameric prohead pRNA (pRNA),^[^ ^34^, ^35^ ^]^ and one ring of gp16 ATPase (Figure 1A). The three rings of the motor operate in concert to translocate dsDNA by a revolving “push‐through one‐way valve” mechanism^[^ ^10^ ^]^ without rotation or coiling of dsDNA^[^ ^3^, ^36^, ^37^, ^38^ ^]^ through the interplay of conformational changes and electrostatic interactions among the motor components. Structurally, the hexameric pRNA ring braces the hexameric ATPase and the dodecameric connector,^[^ ^39^ ^]^ of which the connector ring is docked by the pRNA ring via electrostatic interactions (Figure 1B). The three consecutive basic residues Arg‐Lys‐Arg in the N‐terminus of the connector are required for binding to the interlocking domain of pRNA by electrostatic forces. ATPase binds to the helical domain of pRNA plausibly through electrostatic interactions, for which the evidence is still needed.

The connector is shaped like a truncated cone with the N‐terminal at the narrow end and the C‐terminal at the wider end. The negatively charged dsDNA is attracted to the positively charged lysine rings located along the connector channel (Figure 1B). The gating property of the channel functions possibly in control of the directional dsDNA translocation into the procapsid against the internal pressure of the packaged DNA. The reversal pressure increases the probability of DNA contact with the C‐terminal flexible domain. These contacts induce conformation changes with stepwise gating reduction to avoid dsDNA leakage. Such three‐step conformational changes are common to dsDNA motors of many phages such as T3, T4, SPP1, and Phi29.^[^ ^40^ ^]^ It is also possible for keeping the connector in contact to prepare for ejection during the infection process. The exact mechanism for gating in the one‐way valve function is poorly understood, thus demanding further investigation. Yet, the revolving “push‐through one‐way valve” mechanism entails the separate roles of the connector and the ATPase, both of which are embraced by pRNA into an integrated motor active in DNA translocation through the interplay of conformation alternations and electrostatic interactions among the motor parts (Figure 1B,C).

The gp16 ATPase, geared by pRNA, pushes the viral genome into the procapsid through the connector channel.^[^ ^42^ ^]^ The push motion of dsDNA by gp16 is mediated via the conformational changes of ATPase. Particularly, the pushing‐DNA action involves a transition of the closed DNA‐high affinity conformation (Figure 1B,C) to the expanded low‐affinity conformation of the subunit in the hexameric ATPase ring (Figure 1C). The ATPase conformation changes are allosterically regulated by ATP and dsDNA. ATP binding to ATPase stabilized by electrostatic interaction increases ATPase affinity for the negatively charged DNA plausibly by increasing the positive charging to the DNA binding surface of the ATPase through dielectric allostery (Figure 1B,C). Upon ATP binding, a single subunit that is bridged by the arginine finger to form a transient dimer with its adjacent subunit in the hexameric ATPase (Figure 1A) switches to the closed conformation. The transacting arginine fingers are essential for sequential translocation of the viral DNA via the revolving motion; in fact, the finger functions as the motor starter for translocating dsDNA.^[^ ^43^ ^]^ The subsequent DNA binding to ATPase facilitates ATP hydrolysis that decreases ATPase affinity for the negatively charged DNA, which then repels the DNA substrate (Figure 1C). Upon ATP hydrolysis, the subunit adopts the expanded conformation which lowers its affinity for DNA, and from which DNA is eventually released. The adjacent subunit in the dimer then assumes the closed conformation induced by ATP binding to snatch DNA (Figure 1B) and alternatively takes the expanded conformation to lift DNA up (Figure 1C). In fact, the 3D models of the pRNA–gp16 complex and its conformation change in response to ATP or ADP binding show the closed conformation of the whole complex in the presence of ATP (Figure 1B) but the open configuration during ATP hydrolysis (Figure 1C).^[^ ^44^ ^]^

Further evidence for this model comes from the recent studies that suggest the inchworm revolving mechanism.^[^ ^45^ ^]^ A study combining X‐ray structural determination with molecular modeling and simulations shows a nucleic acid‐binding groove on the protein surface of the C‐terminal domain that is more suitable for binding to pRNA than to DNA.^[^ ^45^ ^]^ It also retains a transient DNA‐binding surface, which may have a role in DNA translocation. There is a dynamic pRNA‐dependent interplay between the N‐ and C‐terminal domains of gp16 important for assembly to drive the transport of DNA into the procapsid plausibly by an inchworm revolving mechanism.^[^ ^45^ ^]^ In this inchworm‐revolution model, the C‐terminal domain of gp16 interacts with pRNA while the N‐terminal contains the ATP binding domain^[^ ^13^ ^]^ to execute the “push‐through one‐way valve” function to translocate dsDNA. The N‐terminal function in this model received more experimental support from an investigation showing that the subunits of the ring ATPase alternate between a dwell state of ATP loading and a burst state of ATP hydrolysis to translocate one turn of DNA.^[^ ^46^ ^]^ Hence, the components of the entire motor act in a dynamic chain action to accomplish the work.^[^ ^41^, ^45^ ^]^

### 2.2 Hexameric ATPases of the viral and cellular dsDNA motors in the prokaryotic and eukaryotic domains

**FIGURE 2:**
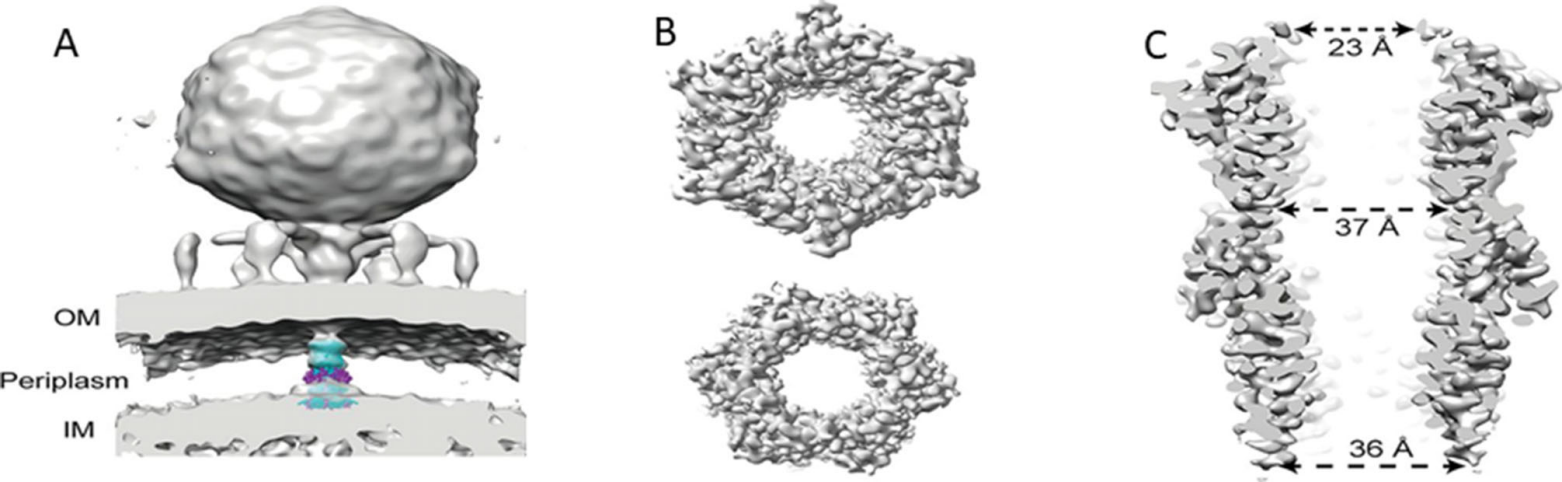
The dsDNA translocation complex of bacteriophage T7 in the periplasmic space of bacteria. (A) Cryo‐electron tomography of the in‐vitro assembled tubular core complex docking into the T7 3D modeling of DNA ejection into bacteria (gp15, light blue; and gp16, purple). Reproduced with permission.^[^ ^25^ ^]^ Copyright 2021, National Academy of Sciences. (B) Top and bottom views indicating the dimensions of the gp15 complex assembly at 3.6‐Å resolution in the cryo‐EM map. (C) A side view showing the dimensions of the internal channel of the gp15 assembly

**FIGURE 3:**
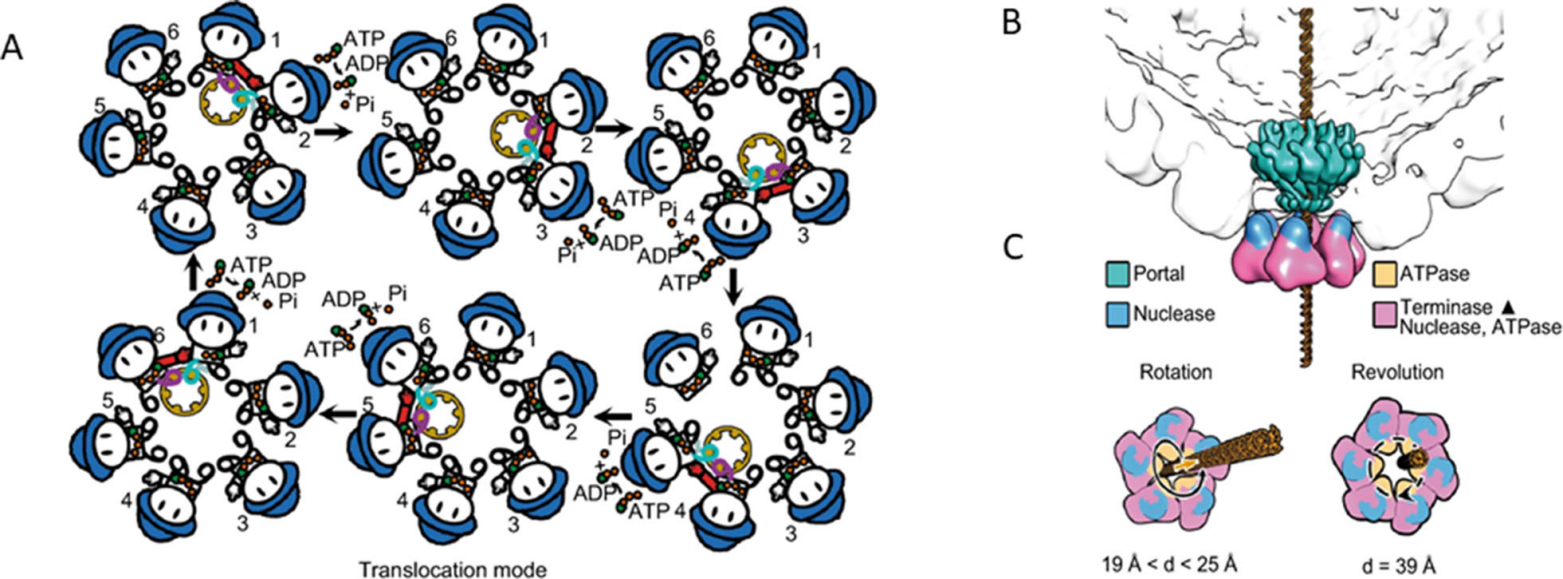
The structure and proposed mechanism of the herpesvirus DNA packaging motor. (A) Top view of the revolving mechanism. (B) The complex of the ATPase. (C) The dimeric subunits bind DNA. The hands, the arginine finger with the red indicating the active state; and the hat, the nuclease that cleaves the concatemeric DNA for packaging of the genome. Reproduced under the terms of the CC‐BY 4.0 license.^[^ ^51^ ^]^ Copyright 2020, The Authors.

**FIGURE 4:**
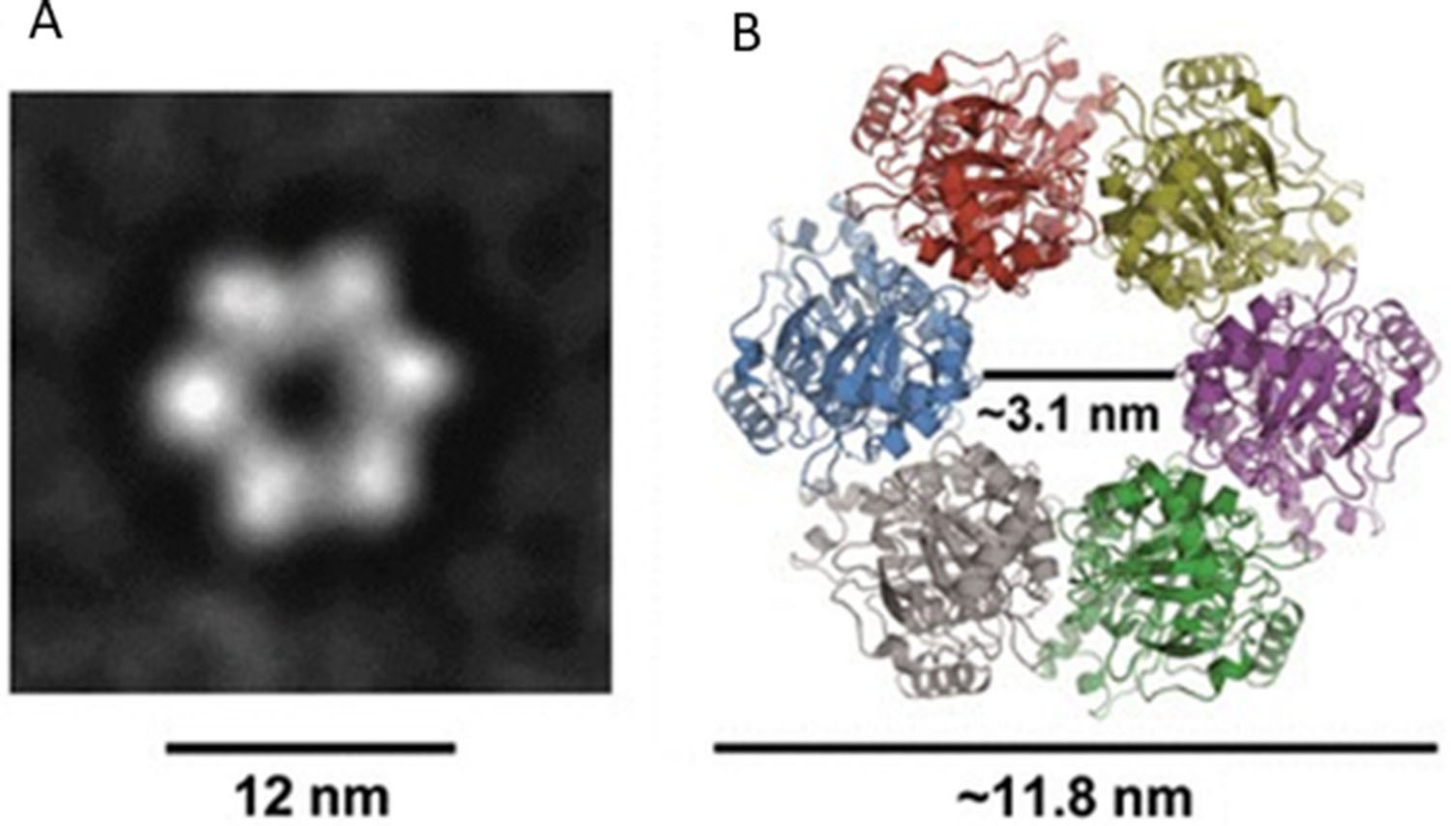
TraBpSVH1 electron microscopy. (A) The hexameric ring‐shaped structure has a central pore with a diameter of ∼12 nm. (B) A pore size is approximately 3.1 nm. Reproduced with permission.^[^ ^26^ ^]^ Copyright 2011, European Molecular Biology Organization

**FIGURE 5:**
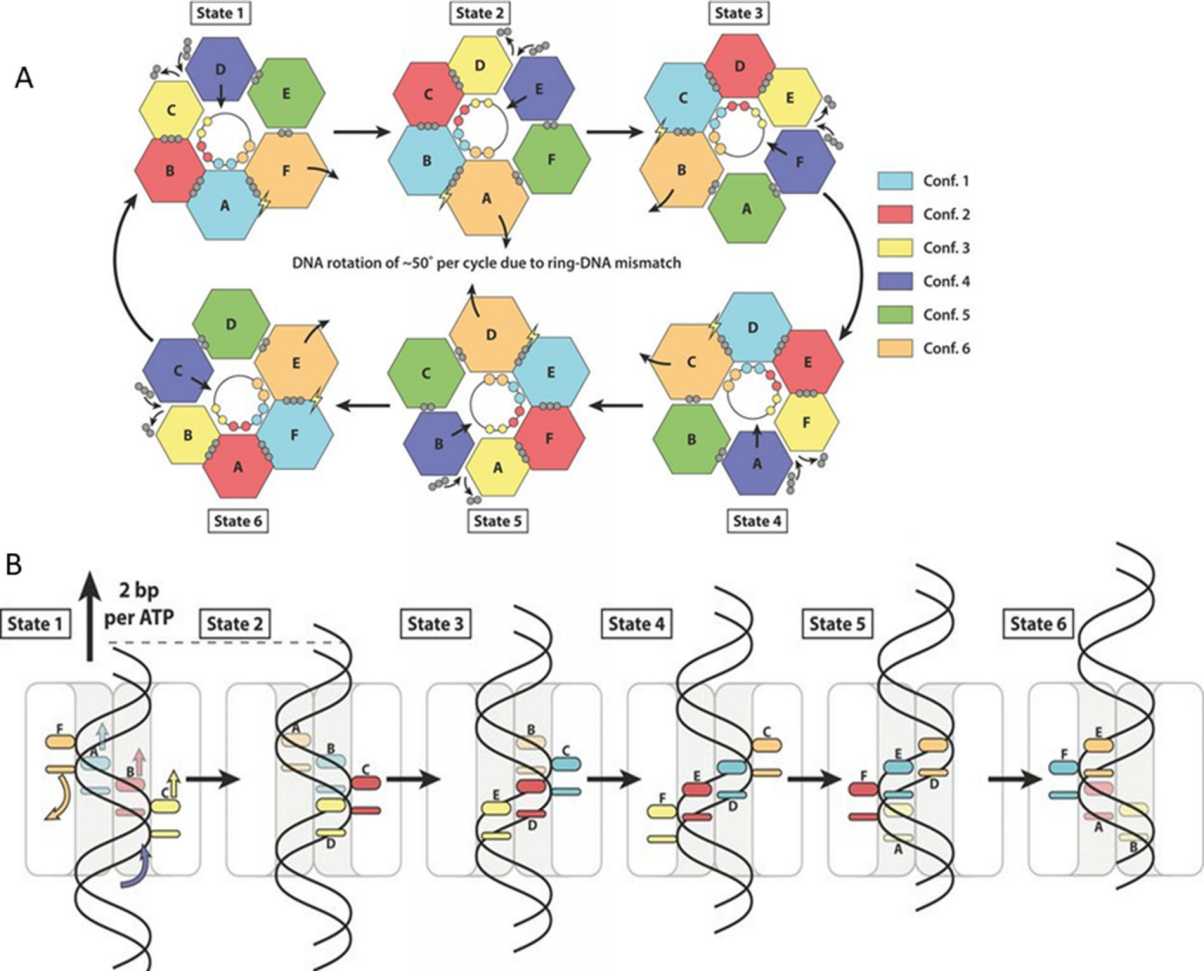
Model for dsDNA translocation by FtsK. (A) A top view of the sequential conformation changes around the hexameric ring. The conformations rotate clockwise around the non‐rotating subunits in the ring. (B) Translocation of the dsDNA through the FtsKαβ pore of the two loops formed by four DNA binding subunits and organized into two spiral staircases per state. Spheres: phosphates; lightning bolt: hydrolysis‐competent state. Reproduced under the terms of the CC‐BY 4.0 license.^[^ ^28^ ^]^ Copyright 2020, The Authors

The parallel architectures of the hexameric ATPases in the DNA motors of the phages (Figures 1 and 2), plasmid‐encoded proteins (Figure 4), herpesvirus (Figure 3), and FtsK (Figure 5) are suggestive of the similar operating mechanisms of the eukaryotic and prokaryotic motors. Bacteriophage T7, like phi29, is a virus that infects the bacterium Escherichia coli.

#### 2.2.1 DNA translocation by herpesvirus terminase complex

Similarly, the genome packaging machinery of the herpesvirus terminase complex shows a hexameric ring assembly of six subunits of heterotrimers with pUL15, pUL28, and pUL33. Among those subunits, pUL15 acts as an ATPase/terminase and forms a hexameric central channel with an internal diameter of 3.9 nm greater than that of dsDNA (Figure 3C). The channel contains the transacting arginine fingers analogous to that of the Phi29 phage (Figure 3A) essential for ATP hydrolysis. Such an analogous architecture to the phage motor suggests that the herpesviral motor translocates DNA in a sequential revolving motion (Figure 3B).^[^ ^29^ ^]^

#### 2.2.2 DNA translocation by TraB of bacterial plasmids

Like the bacteriophage DNA motors and bacterial FtsK, some bacterial plasmid‐encoded proteins and cellular motors show the hexameric ring structures for DNA translocation. TraB mediates bacterial plasmid conjugation by specifically binding to the repeated 8‐bp motifs on the conjugative plasmid through the helix α3 of the C‐terminal winged‐helix‐turn‐helix domain. TraB forms a hexameric ring as revealed by an EM‐based structural analysis.^[^ ^52^ ^]^ It embeds into the lipid bilayers (Figure 4A) with an internal diameter of ∼3.1 nm (Figure 4B) to accommodate dsDNA.^[^ ^26^ ^]^ Formation of four TraB hexamers is plausibly a prerequisite for translocation of genome across cell membranes.^[^ ^52^ ^]^

#### 2.2.3 FtsK of bacterial dsDNA translocase

Highly conserved among all bacteria,^[^ ^53^ ^]^ the FtsK protein has the motor portion located at the C terminus.^[^ ^54^ ^]^ This biomotor is made up of three parts: α, β, and γ. The α and β are involved in hexamer formation and hydrolyzing ATP,^[^ ^54^ ^]^ and the γ domain is responsible for binding to specific sequences of DNA called FtsK‐orienting polar sequences (KOPS).^[^ ^55^ ^]^ When no DNA is present, FtsK exists as a monomer. In the presence of dsDNA, the α and β domains assemble into hexameric rings with an outer diameter of ∼120 Å. The inner channel of the FtsK hexamer has a diameter of ∼30 Å,^[^ ^54^, ^56^ ^]^ which is much wider than the diameter of ds DNA helix (∼20 Å). The native FtsK motor protein used in biochemical studies was not well‐suited for structural work due to its low solubility; so a soluble mutant was created. Negative stain transmission electron microscopy and analytical ultracentrifugation were used to show that this soluble mutant existed as monomers in the absence of DNA but oligomerized into hexamers when combined with dsDNA.^[^ ^56^ ^]^ Additionally, X‐ray crystallography shows that the β portion was similar in structure to other oligomeric ATPases. The X‐ray crystal structure of the hexamer exhibits that each monomer had its own active site, in contrast to other hexameric motors where the active site is located between two monomers.^[^ ^56^ ^]^ Interestingly, electron microscopy revealed that the FtsK motor was able to form double rings around strands of DNA, with the rings aligned β to β.^[^ ^56^ ^]^ The significance of the double rings is currently unknown.

The combination of X‐ray crystallography and electron microscopy provides clues on the mechanism for the FtsK motor. The X‐ray crystal structures of the FtsK monomer show the α and β domains acting as a hinge. When the hinge opens, the α domain moves 5.5 Å away from β, which correlates to 1.6 base pairs of dsDNA. Based on this observation, Massey et al. suggested that FtsK translocates dsDNA in a rotary inchworm mechanism. In this model, DNA first binds to the α domain. ATP binding and hydrolysis initiate a conformational change that allows DNA to bind to β and opens the hinge to move α away from β. As the hinge opens, the DNA is pulled through the channel. At the end of the catalytic cycle, the DNA remains bound to β and is completely disengaged from α. Since the DNA is helical, the same α domain will not be able to bind the DNA again, so another monomer goes through the catalytic cycle. In this way, all 6 monomers work together to drive DNA through the channel in a revolving manner.^[^ ^56^ ^]^

FtsK assists in resolving the chromosomal dimers by translocating the chromosome duplicates during cell division. In the process of dsDNA translocation, the Cryo‐EM imaging of the domain of FtsK motor shows a ring structure of the homo‐hexamers, of which each subunit adopts a special conformation and nucleotide states (Figure 5A).^[^ ^28^ ^]^ The two DNA‐binding loops made of the four subunits in the hexameric ring form a pair of spiral staircases holding the two DNA strands inside the ring (Figure 5B). From the loops‐dsDNA interactions, conformations of the ATPase domains appear to change simultaneously at each catalytic step likely to create the motion of the DNA substrate in a filament treadmilling‐like fashion (Figure 5B). It is impossible for the entire bacterial chromosome to rotate during translocation through the internal diameter of the FtsK hexameric ring that is larger than the external diameter of dsDNA. Therefore, the hexameric ATPases of these viral and cellular dsDNA motors exhibit parallel architectures, based on relevant revolving functions that operate these motors for dsDNA translocation.

## 3 INTERPLAY BETWEEN MOTOR FORCE GENERATION AND CONFORMATIONAL CHANGES

The parallel mechanisms of the viral and cellular dsDNA motors involve interaction between force generation and conformational alterations of the motor components for DNA translocation. Similar to other biological activities, the force generation is powered by energy released from ATP hydrolysis by the ATPase domain.^[^ ^42^, ^60^, ^61^, ^62^, ^63^ ^]^ DNA translocation by the phi29 DNA packaging motor has been generally believed to convert the chemical energy stored in ATP into the physical motion of DNA.^[^ ^64^, ^65^, ^66^ ^]^ The energy conversion is achieved through ATPase conformation changes from high to low affinity for DNA so that energy is transformed into DNA motion via alternation of high‐low affinities of ATPase for DNA. Since the phi29 ATPase is hexameric, the DNA translocation by this motor is a sequential action of six ATPase subunits that are coordinated in a “monomer‐dimer” conformation by an arginine finger. Such sequential conformational changes have been also found in FtsK while translocating dsDNA.^[^ ^28^ ^]^ The phi29 motor consists of ATPase, connector, and prohead RNA (pRNA), of which pRNA not only braces the ATPase and the connector but also plays a role in motor conformation dynamics. This section discusses the interplay between the force generation and the resultant conformational changes of the motor components that drive DNA translocation.

### 3.1 ATPase conformation change is triggered by ATP binding without ATP hydrolysis

**FIGURE 6:**
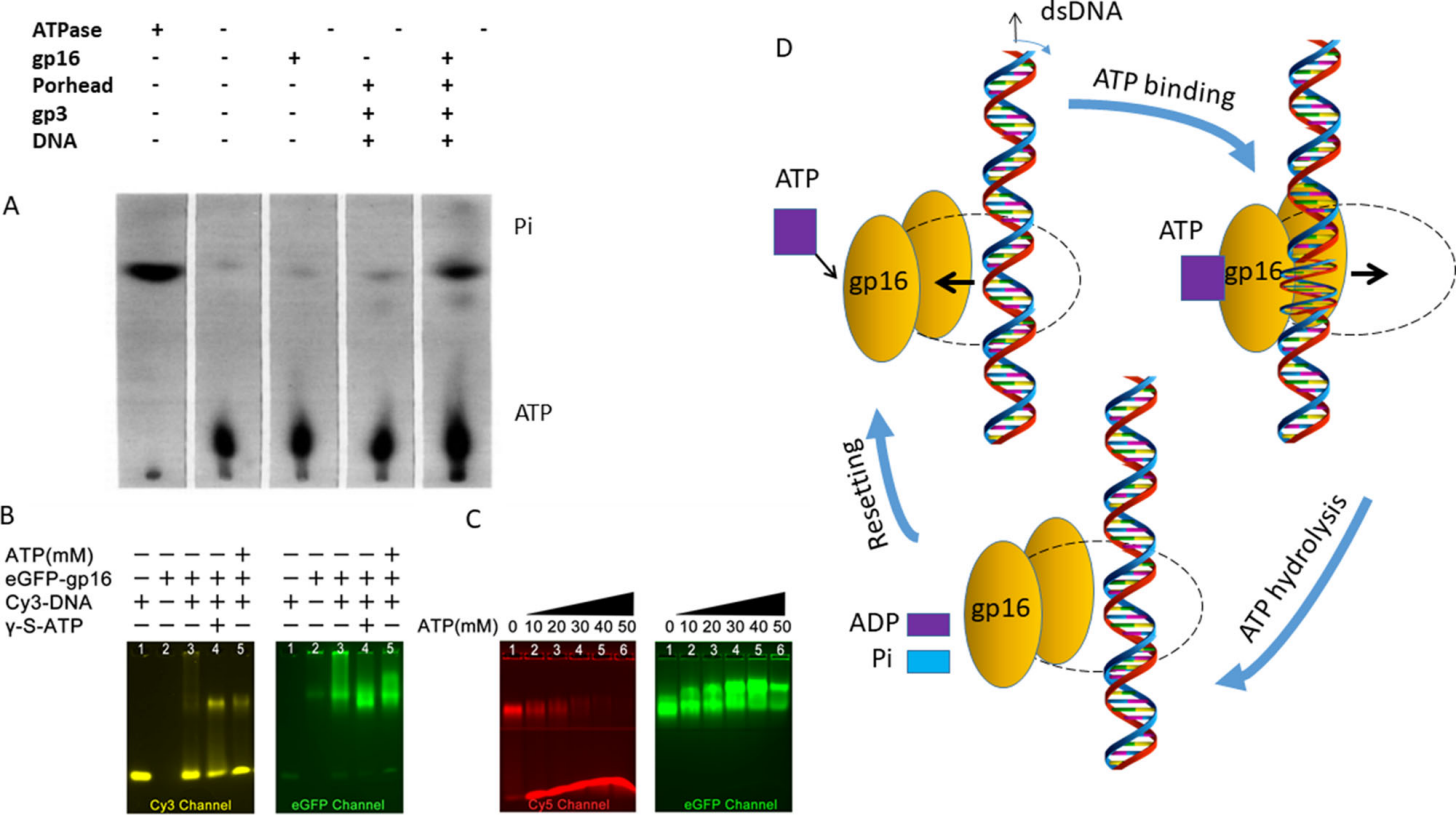
ATPase conformation change triggered by ATP binding without ATP hydrolysis. (A) A chromatogram displaying ATP hydrolysis during DNA‐gp3 packaging. (Adapted with permission.^[^ ^60^ ^]^ Copyright 1987, Elsevier. (B) EMSAs showing the binding of DNA to gp16 in the presence of ATP or γ‐s‐ATP. Reproduced with permission.^[^ ^15^ ^]^ Copyright 2013, American Chemical Society. (C) When an increased concentration of ATP was added into DNA/gp16/ γ‐s‐ATP complex, more DNA was able to be released. Reproduced under the terms of the CC‐BY 4.0 license.^[^ ^76^ ^]^ Copyright 2016, The Authors. (D) A schematic interpretation of the data

The conversion between chemical energy and physical motion encompasses ATP binding to the ATPase, conformational changes of the ATPase, ATP hydrolysis by the ATPase, and translocation of DNA. Sequence alignment of ATPases shows that both the A‐ and B‐type consensus sequences, termed Walker A and Walker B for ATP binding and ATP hydrolysis, respectively, exist in the motor ATPases of FtsK in bacteria and bacteriophages. The phage families include phi29, SPP1, P22, T7, and HK97.^[^ ^60^, ^67^, ^68^, ^69^ ^]^ As the conformational change of some biomotors was found to be associated with ATP binding without ATP hydrolysis,^[^ ^64^, ^65^, ^66^, ^70^, ^71^ ^]^ the process of conversion of chemical energy into motion by the biomotors is still a conundrum. The discovery of the DNA‐dependent activity of the phi29 motor ATPase gp16 of the hexameric AAA+ family^[^ ^60^ ^]^ (Figure 6A) has shed light on this dark box. During its interaction with ATP, gp16 undergoes a cycle of conformational alterations; in fact, it switches between conformations of either high or low affinity for dsDNA. After ATP binding, the enzyme undergoes a conformational alteration and subsequently increases its affinity for DNA, tightly clinching dsDNA. One ATP molecule is adequate to create the high affinity state for DNA in the ATPase ring. After ATP hydrolysis, it loses the affinity, pushing dsDNA away. Therefore, forces are generated as ATP binding to ATPase, and subsequent hydrolysis of ATP induces the conformational changes of ATPase. Chemical and binding energies are transformed into DNA motion via the gain and loss of affinities of ATPase for DNA.

Conceivably, during the ATP‐ATPase interaction, chemical energy in ATP is transformed into physical motion through conformation changes of ATPase for the phi29 DNA packaging as studied with several assays. One of these assays is the electrophoretic mobility shift assay (EMSA),^[^ ^42^, ^72^ ^]^ the results of which suggest that the ATPase experiences conformational alterations upon its binding and hydrolysis of ATP, accompanied by high or low affinity of ATPase to DNA.^[^ ^3^, ^42^ ^]^ In EMSA with the ATPase labeled by eGFP without affecting its function or activity,^[^ ^73^ ^]^ the qualitative data demonstrate that in the absence of ATP, ATPase gp16 barely binds to dsDNA. However, in the presence of γ‐S‐ATP, a non‐hydrolyzable ATP analog, the gp16/dsDNA complex is stalled and unable to release DNA^[^ ^42^ ^]^ (Figure 6B, lanes 4 and 5). Moreover, in the presence of γ‐S‐ATP, there is an augmentation in energy transfer from eGFP‐gp16 to Cy3‐dsDNA compared to its absence as indicated by Forster resonance energy transfer analysis. When γ‐S‐ATP is present, the gp16‐dsDNA complex becomes abundant, as shown by an overlap wavelength spectrum in the eGFP‐gp16 and cy3‐dsDNA indicated in a sedimentation study utilizing a 5%–20% sucrose gradient. In the presence of ATP or γ‐S‐ATP, the gp16 ATPase band shifts to a new position in EMSA (Figure 6B, lanes 3, 4, and 5). These data suggest that given γ‐S‐ATP is non‐hydrolyzable, the new ATPase conformation is induced by ATP binding without hydrolysis. The conformational alteration induced by ATP binding is also evidenced by a Walker A motif mutant phenotype deficient in both ATP binding and conformation induction.^[^ ^60^, ^74^ ^]^ The functional assays identified a mutation in the Walker A motif^[^ ^72^ ^]^ responsible for ATP binding^[^ ^75^ ^]^ and confirmed the lack of conformational change by the mutation.^[^ ^3^, ^60^, ^74^ ^]^ Another mutation in gp16 Walker B motif abolished the catalytic step.^[^ ^72^ ^]^ The results from both EMSA and mutation assays support the conclusion that the viral ATPase conformation change is triggered by ATP binding without ATP hydrolysis.

### 3.2 ATP hydrolysis and Pi release causing phage ATPase conformational reversion

In the phi29 biomotor, gp16 is an ATPase dependent on DNA (Figure 6A).^[^ ^60^, ^77^, ^78^ ^]^ The activity of this ATPase provides energy to the motor through ATP hydrolysis into ADP and inorganic phosphate.^[^ ^42^ ^]^ Initially, the gp16 ATPase binds DNA in the presence of ATP or γ‐S‐ATP as shown by EMSAs (Figure 6B). In the presence of increasing concentrations of ATP, the DNA/gp16/ γ‐S‐ATP complex disappears while the gp16 band remains at the highest ATP concentration (Figure 6C). This result indicates that the DNA substrate is released when ATP is present. As dsDNA is released from gp16, ADP has less of an effect than ATP, and AMP has no effect^[^ ^3^ ^]^ as demonstrated by EMSAs and sucrose gradient sedimentation. The release mechanism is further investigated with the gp16/dsDNA/γ‐S‐ATP complex purified by sucrose gradient for an ATP hydrolysis kinetic assay. After the addition of ATP to the purified complex, hydrolysis of ATP to ADP and inorganic phosphate occurs. This result confirms the hydrolysis activity of the purified gp16/dsDNA/γ‐S‐ATP. Upon ATP hydrolysis,^[^ ^42^ ^]^ gp16 dissociates from dsDNA. These data demonstrate that after ATP hydrolysis, gp16 undergoes a further conformational alteration that pushes dsDNA away from the motor complex^[^ ^76^ ^]^ (Figure 6D). All these results suggest that ATP hydrolysis causes the release of dsDNA from gp16 through the conformation changes of the ATPase that drive the DNA substrate to depart from the interior pocket of the ATPase. The conformational alteration of the ATPase is thereby translated into the physical motion of genomic DNA toward the next subunit of the motor (Figure 6D) and ultimately into the viral capsid. Altogether, the revolving motion of dsDNA is driven by forces generated from a combination of both ATPase activity and conformational changes of the enzyme.

### 3.3 Regulation of the sequential action of the ATPase by an arginine finger

Since the phi29 dsDNA motor consists of multiple subunits, DNA translocation requires communication among the motor components. As the arginine finger motif and the Walker A and B motifs are commonly found in the ASCE ATPases,^[^ ^79^, ^80^, ^81^, ^82^, ^83^, ^84^, ^85^, ^86^, ^87^, ^88^, ^89^ ^]^ it was hypothesized that these components were coordinated by the arginine finger. The rationale for this hypothesis was that the arginine residue was situated in the active ATPase ring proximal to the γ‐phosphate of the bound ATP^[^ ^81^, ^84^, ^85^, ^86^ ^]^ and involved in the formation of the ATP binding pocket.^[^ ^83^, ^86^, ^87^, ^88^, ^89^ ^]^ Testing of this hypothesis^[^ ^90^ ^]^ led to the results showing that the arginine finger motif played an important role in coordinating the various subunits of the ATPase for dsDNA translocation.^[^ ^76^ ^]^ This motif is essential for the function of the ATPase because mutations inactivating the arginine finger abrogated ATPase oligomerization, ATP binding/hydrolysis, and DNA translocation. Furthermore, the arginine finger played a role in the noncovalent dimer formation of the ATPase subunits by bridging the monomers (Figure 1A). Interestingly, the isolated dimers alone had no DNA translocation activity. The activity was restored by the addition of monomers. These results suggested that the hexameric ATPase ring consisted of both dimers and monomers, both of which were required to form the asymmetrical hexameric organization. Moreover, this asymmetrical hexameric model of phi29 ATPase was supported by structural evidence of many other ATPases that contain one noncovalent dimer and four monomer subunits.^[^ ^91^, ^92^, ^93^, ^94^, ^95^, ^96^ ^]^ Functionally, the arginine finger promotes dimer formation inside the hexamer, thereby regulating the sequential action of the ATPase ring in the DNA packaging motor^[^ ^76^ ^]^ likely by two distinct regulatory mechanisms.^[^ ^97^ ^]^ In the first mechanism, the catalytic activity of gp16 of a single subunit is up‐regulated by dsDNA, transforms the ATPase through the arginine finger into a global regulator that induces the hydrolysis and the nucleotide exchange phases. In the second mechanism, the arginine finger in each subunit of gp16 stimulates the activities of ATP hydrolysis and ADP‐ATP exchange in its neighboring subunit. These mechanisms are possibly fundamental to intersubunit coordination of ATPases, commonly seen in the other ring ATPases^[^ ^97^ ^]^ such as FtsK. FtsK operates by a treadmilling mechanism, through which four subunits work together transiently (Figure 5). The arginine finger helps with the communication between the subunits.^[^ ^28^ ^]^

### 3.4 The role of pRNA in motor conformational dynamics

**FIGURE 7:**
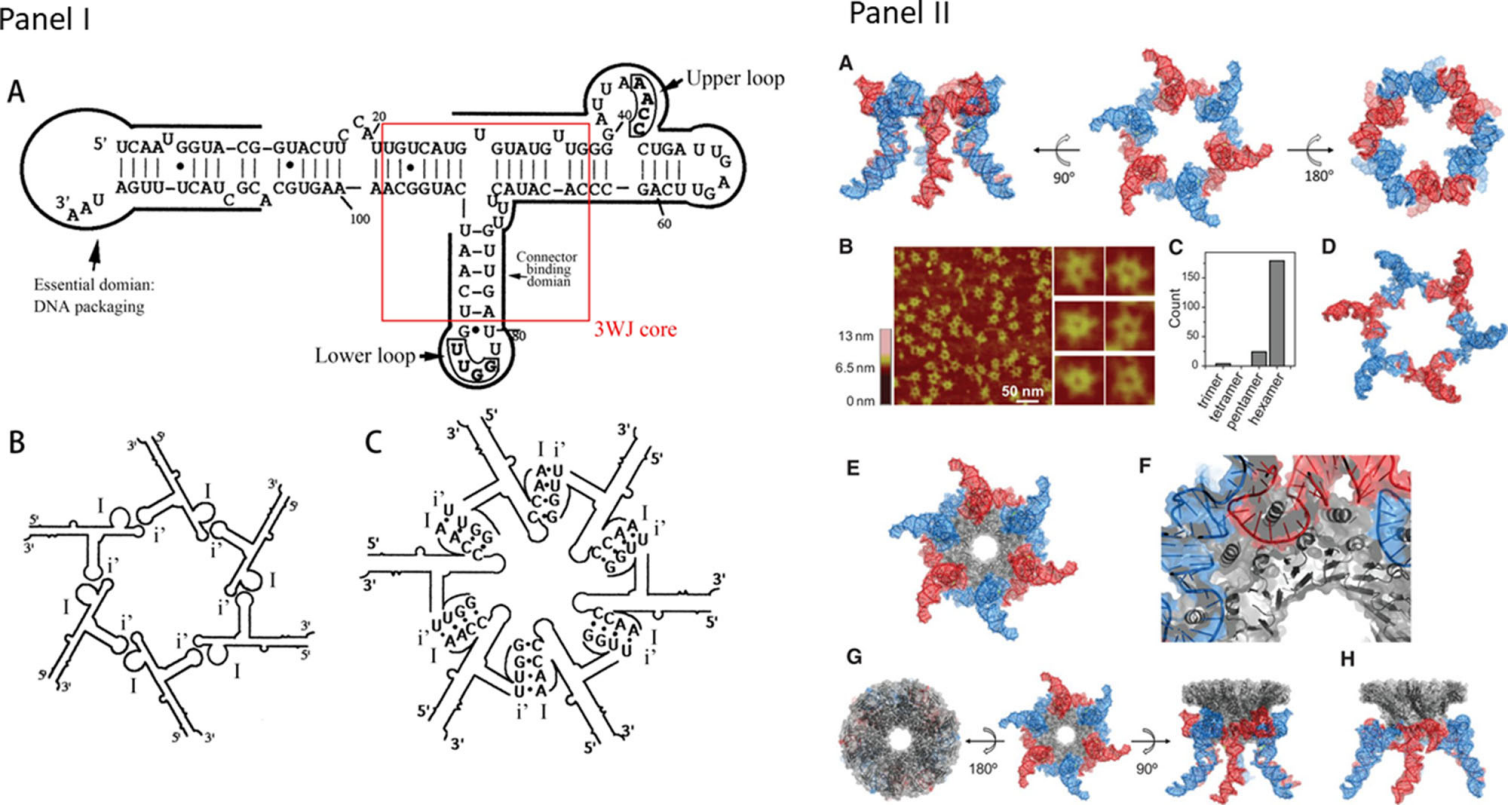
Structures of hexameric pRNA and the association with the connector. Panel (I): The secondary structures of pRNA and the hexamers. (A) The 5′/3′secondary structure of pRNA with the 3WJ shown in the boxed region. The 3WJ connects the helical domain to the interlocking procapsid binding domains. Reproduced under the terms of the CC BY‐NC 4.0 license.^[^ ^114^ ^]^ Copyright 2016, The Authors. (B,C) Two conformations of the pRNA hexametric rings by the upper or lower loop interaction. Reproduced with permission.^[^ ^115^ ^]^ Copyright 1998, Cell Press. Panel (II): The hexameric pRNA ring and how it connects to the dodecameric connector channel of phi29. (A) Different views of the pRNA hexamer based on the crystal structure of 3WJ. (B) Atomic force microscopy (AFM) images Illustrating the hexameric nature of the pRNA complex. (C) Histogram showing the distribution of the stoichiometry of RNA particles as determined by AFM. (D) Top view of a computational model of the published 3D computer model of hexameric pRNA generated based on biochemical data (PDB 1L4O).^[^ ^111^ ^]^ (E) Model illustrating how the pRNA hexamer binds to the dodecameric connector ring based on the 3WJ and connector crystal structures (PDB 1H5W)^[^ ^116^ ^]^ crystal structures. (F) A magnified view showing how the N‐terminal helices of the connector interact with pRNA. (G) Different views of the hexameric pRNA‐connector assembly. (H) A side‐view of the 3D model of the hexameric pRNA‐connector assembly based on biochemical data (PDB 1L4P).^[^ ^117^ ^]^ Reproduced under the terms of the CC BY‐NC 3.0 license.^[^ ^111^ ^]^ Copyright 2013, The Authors

The fact that the operation of the phi29 motor relies on Mg^2+^ ions suggests a structural factor intrinsic to this motor–pRNA, joining the connector and ATPase domains.^[^ ^98^, ^99^ ^]^ This conserved pRNA is a non‐coding RNA component that is necessary for viral genome translocation and packaging.^[^ ^34^, ^100^ ^]^ Its secondary structure consists of 117 nucleotides (nt) folded into a complex structure containing two major domains (Figure 7IA); a helical domain with an open 5′/3′ end that binds to gp16,^[^ ^101^ ^]^ and an interlocking domain with stems and loops that binds to the connector.^[^ ^102^, ^103^, ^104^, ^105^, ^106^, ^107^ ^]^ A thermostable three‐way junction (3WJ) motif^[^ ^108^, ^109^ ^]^ is located in the center and branches out to hold the gp16‐binding domain and the connector‐binding domain of the right‐hand and left‐hand loops (Figure 7IA). Intermolecular interactions of these loops form complex structures, including dimeric, tetrameric, and hexameric rings^[^ ^110^ ^]^ (Figure 7IB,C, and IIA–D). This pRNA 3WJ shows an exceptional thermodynamic stability to support the packaging motor.^[^ ^108^, ^109^, ^111^ ^]^ Crystal structure of pRNA 3WJ motif also indicates the divalent ion‐promoted thermostability, with which pRNA binds to procapsid with the portal connector serving as the foothold (Figure 7IIE–H).^[^ ^111^ ^]^

The specificity and stringency of dsDNA translocation by the motor were determined by 5′/3′ proximal end that restricted the packaging of a single DNA fragment with a fixed orientation.^[^ ^112^, ^113^ ^]^ These observations point to a role that pRNA may play in dsDNA translocation. Actually, pRNA and gp16 form a complex, and pRNA affects the complex conformation. The role of pRNA becomes clear when observing the 3D models of the gp16‐pRNA complex and how its conformation changes depending on whether it is bound to ATP or ADP. These models were established by using a combination of biotechnology, biochemistry, mutation, deletion,^[^ ^101^, ^104^ ^]^ crystallography, small angle X‐ray scattering, and chemical probing.^[^ ^44^ ^]^ The model shows that gp16 binds to the 5′/3′ proximal end of pRNA specifically.^[^ ^101^, ^104^ ^]^ The pRNA‐gp16 complex takes a ‘Z’‐shaped conformation. Interestingly, in the presence of ATP, the complex assumes a closed conformation. Upon ATP hydrolysis, the gp16‐pRNA hinges open. The open conformation remains until ADP is released, and then the domain is reset to the relaxed conformation.^[^ ^44^ ^]^ This structural evidence suggests that upon ATP binding, the gp16‐pRNA complex in a closed conformation binds DNA. The subsequent ATP hydrolysis opens up the complex to release DNA.^[^ ^44^ ^]^ Taken together, these studies show that ATP interacting with gp16 changes the conformation of the pRNA‐gp16 complex, and this conformation change drives DNA translocation.

Altogether, the main function of pRNA is to maintain the structural integrity of the connector and ATPase portions of the phi29 motor. The integrity of these portions is strengthened by the two domains of pRNA. The 3WJ (Figure 7IA) holding together the two domains has direction‐dependent mechanical stability that provides a framework for the connector and the ATPase. The interlocking domain of pRNA is associated with the connector and head shell proteins. The helical domain of pRNA is associated with gp16 that generates force for dsDNA translocation. It may play a role in facilitating conformation changes and force generation in gp16. Since the ATPase‐binding domain restricts the packaging of a single DNA fragment with a fixed orientation,^[^ ^112^, ^113^ ^]^ this domain with the ATPase may restrict the backward motion of dsDNA and multiple DNA fragments.

## 4 ROLE OF ELECTROSTATIC INTERACTIONS IN DNA TRANSLOCATION

The parallel mechanisms of the viral and cellular dsDNA motors illustrate the role of electrostatic interactions in the translocation of dsDNA. Among the dsDNA motors, dsDNA translocation by the phi29 motor is mediated by conformation changes most likely via the electrostatic interactions among the motor components, such as ATPase, connector, and pRNA. Through electrostatic interactions, the dodecameric connector is docked with the hexameric pRNA. The stepwise translocation of dsDNA through the connector is mediated by electrostatic forces. The connector exhibits the stepwise gating behavior during genome packaging to avoid reverse motion of dsDNA or leakage. Electrostatic potentials are involved in the force generation of the hexameric ATPase in response to ATP binding and hydrolysis. The interaction of the motor with DNA is also electrostatic in nature. This section discusses the role of electrostatic interactions among the motor components in the context of DNA translocation.

### 4.1 Docking of the hexameric pRNA with the dodecameric connector involves electrostatic interactions

As discussed earlier, the interlocking domain of pRNA holds the connector protein gp10 of the phi29 motor through electrostatic interactions (Figure 7IIE–H). The connector has the wider domain containing the C‐terminus encapsulated inside the procapsid^[^ ^118^, ^119^, ^120^, ^121^ ^]^ whereas the narrow domain housing the N terminus remains flexible and structurally disordered with the 14 residues critical for binding of pRNA.^[^ ^122^, ^123^ ^]^ Moreover, the connector‐pRNA binding involves electrostatic interactions.^[^ ^124^ ^]^ When 25 amino acid residues were deleted or 14 residues were extended from the C terminus, the procapsid assembly was not affected; but if 42 amino acids were added to the N terminus, the DNA‐packaging efficiency was significantly reduced without interfering with the procapsid assembly. Cleaving the extended region returned DNA‐packaging activity.^[^ ^124^ ^]^ These results demonstrate that the integrity of N‐terminus is essential for DNA packaging. Particularly, the arginine and lysine residues in the N‐terminal region are crucial for DNA packaging because replacing them, while not affecting assembly of the procapsid head, completely abolished pRNA binding to the connector and interfered with DNA packaging. Apparently, the consecutive Arg‐Lys‐Arg mediates pRNA‐connector protein binding possibly via electrostatic interactions, similar to how several RNA‐binding proteins including Tat peptide^[^ ^125^, ^126^ ^]^ or poly Arg^[^ ^127^ ^]^ bind polyanionic nucleic acids through interaction with the cationic side groups arginine or lysine. In fact, the involvement of the electrostatic interaction is supported by the results of the procapsid‐RNA binding assay. It tested the mutant procapsid in which the 14–amino acid sequence at the N‐terminal domain of gp10 was replaced with an uncharged peptide. The results indicate no binding between the mutant procapsid with pRNA.^[^ ^124^ ^]^ Therefore, the three consecutive basic residues Arg‐Lys‐Arg in the N‐terminus are required for pRNA binding via electrostatic interactions.

### 4.2 Stepwise translocation of dsDNA due to electrostatic interactions

**FIGURE 8:**
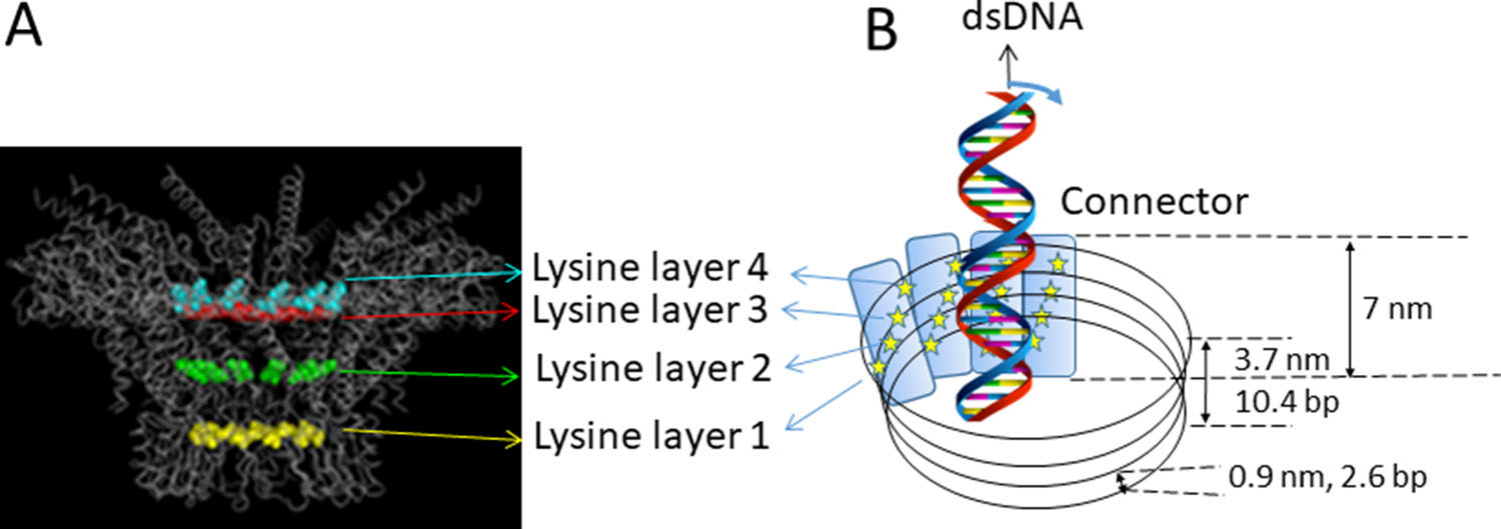
Illustrated is the stepwise translocation of dsDNA revolving along the inner wall of the phi29 connector channel. (A) Side view of the connector showing the four lysine rings arranged inside the inner wall of the connector. Reproduced with permission.^[^ ^15^ ^]^ Copyright 2013, American Chemical Society. (B) The process of the stepwise dsDNA translocation. The negatively charged dsDNA phosphate backbone is attracted to each of four positively charged lysine (stars) rings embedded in the negatively charged connector subunits (vertical bars). The individual lysine ring space and total width are indicated (nm) together with the length of DNA (bp) accommodated. Based on the crystal structure, the length of the connector channel is 7 nm^[^ ^118^, ^120^, ^133^ ^]^ and the height of four lysine layers is about 3.7 nm.^[^ ^118^ ^]^ Each of the lysine layers is spaced at approximately ∼0.9 nm. Given that B‐type dsDNA has a rise of 0.34 nm/bp, 0.9 nm/0.34 nm·bp^−1^ = ∼2.6 bp, which interacts with each lysine ring. For 4 rings, 2.6 bp × 4 = ∼10.4 bp, which is close to a pitch/turn of dsDNA helix 10.5 bp

The viral dsDNA is translocated at varying speeds in many dsDNA bacteriophages. Pauses during the process of dsDNA translocation have been reported in both phi29^[^ ^128^, ^129^ ^]^ and T4.^[^ ^130^ ^]^ These pauses cause stepwise translocation of dsDNA, in which four steps of dsDNA motion have been observed in the phi29 motor^[^ ^128^, ^131^ ^]^ by laser trap experiments (Figure 8). These four pauses may arise from the existence of four ATP hydrolysis associated states of the ATPase; however, a more plausible explanation may arise from the electrostatic interactions between the positively charged amino acids along the motor channel and the negatively charged phosphates in dsDNA (Figure 8).^[^ ^37^ ^]^ Such interactions between the lysine layers of the connector and the phosphate backbone of the dsDNA result in uneven steps in dsDNA translocation due to the mismatch between 10.5 bases per DNA helical pitch and the 12 subunits connector channel wall as reviewed recently.^[^ ^132^ ^]^ The crystal structure of the connector shows 48 positively charged lysine residues present on the overall negatively charged interior channel (Figure 8).^[^ ^118^ ^]^ These residues are arranged as four lysine rings encircling the 12 subunits of the connector. As dsDNA revolves 360° through the 12 connector subunits, the negatively charged phosphate backbone is attracted to the positively charged lysine rings,^[^ ^3^, ^36^, ^37^ ^]^ leading to four pauses or uneven speed during dsDNA translocation.^[^ ^36^, ^37^, ^129^ ^]^ Since the motor channel is overall negatively charged, dsDNA with the same charge is repelled during translocation; for dsDNA to revolve, the positively charged lysines in the channel drag dsDNA to the wall regularly so that the revolving motion of dsDNA is kept on track.

### 4.3 Reversible conformation changes of the motor channel gating

**FIGURE 9:**
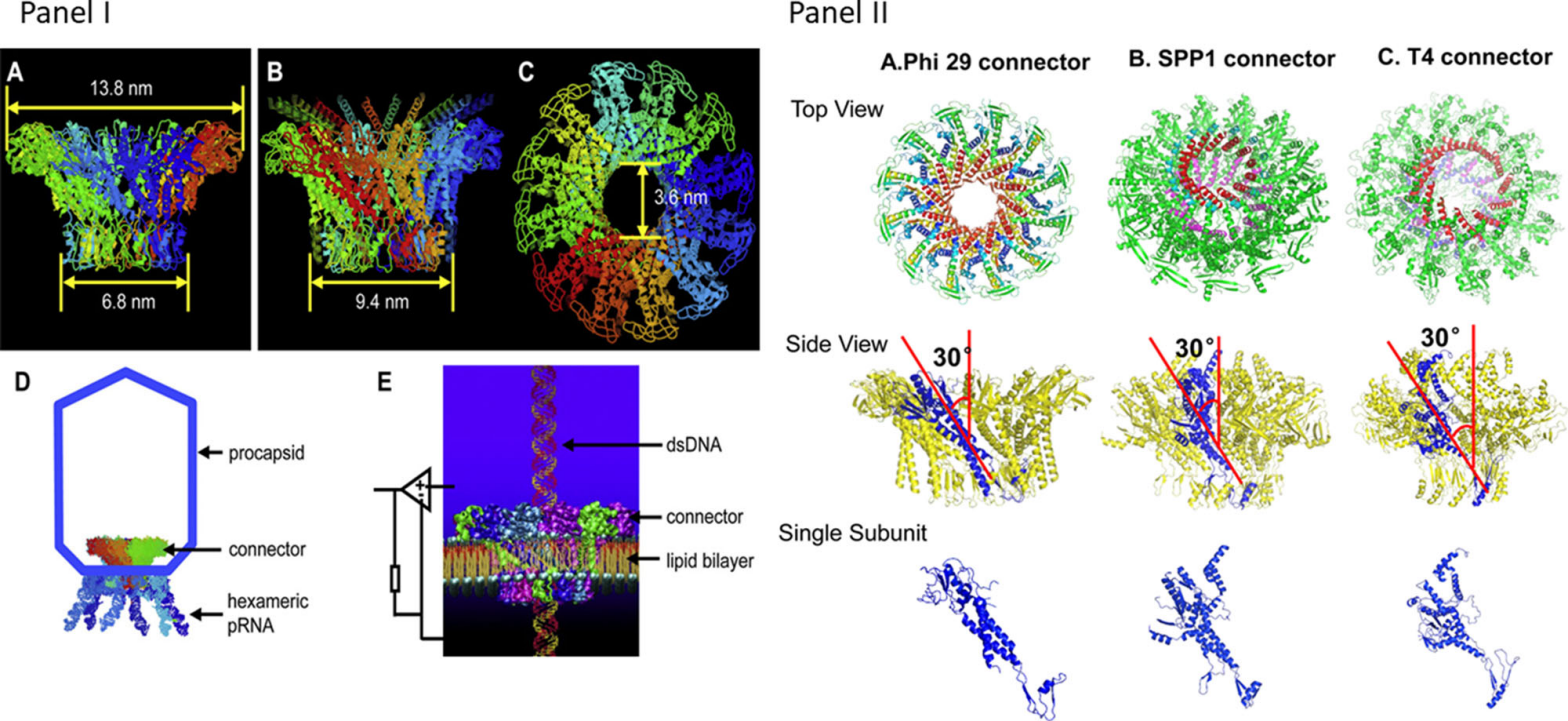
The connector channel structures of the related phages. Panel (I): The phi29 connector structure. (A) Side view excluding the C‐ and N‐terminal fragment; (B) Side view and (C) bottom view with complete protein sequence; (D) Model showing the phi29 DNA packaging motor within the procapsid; (E) Illustration of dsDNA translocation through a connector channel reconstituted in an artificial lipid bilayer to measure conductance. Reproduced with permission.^[^ ^135^ ^]^ Copyright 2011, Elsevier. Panel (II): Structures of different dsDNA bacteriophage portal channels. Top view, side view, and single subunit of (A) phi29; (B) SPP1; and (C) T4 portal protein. Phi29 gp10 PDB: 1FOU; SPP1 gp6 PDB: 2JES; T4 gp20 PDB: 3JA7. Reproduced with permission.^[^ ^40^ ^]^ Copyright 2016, Elsevier

Many protein pores and ion channels use gating to regulate ion transportation through cell membranes. Gating is controlled in different ways, for example, ligand‐gated ion channels respond to the binding of chemical signals to open or close, and voltage‐gated ion channels respond to electrical potential differences near the channel for activation.^[^ ^134^ ^]^ The channel gating of the phi29 DNA packaging motor has been studied, displaying three reversible discrete steps.^[^ ^135^ ^]^ In particular, the orientation of the connector in the membrane was determined to ensure the one‐way traffic mechanism for translocation of dsDNA in the proximal orientation (Figure 9ID,E).^[^ ^136^ ^]^ The C‐terminal side of the connector seems like a trigger for the gating since the addition of the anti‐His tag antibody specific for the His‐tagged C‐terminal connector caused six discrete 31% current reduction steps for the connector. Similarly, the channel current was reduced by ∼31% when a Ni‐NTA nanogold particle was bound to the His‐tagged C‐terminus, displaying the discrete stepwise closing of the channels. Furthermore, three‐step channel conformational changes have been found common to DNA packaging motors of other bacterial viruses T3, T4, SPP1, and Phi29 (Figure 9II).^[^ ^40^ ^]^ Specifically, under a positive voltage in the procapsid‐proximal orientation (Figure 9I D,E), a three‐step gating occurs to the four portal channels of phi29, SPP1, T4, and T3, exhibiting a decrease in electrical current of 33%, 66%, and 99% for each of three steps. In contrast, a three‐step gating of the current increase occurs under a negative voltage as the electric charges are reversed. As the membrane voltage results in electrostatic forces that influence the voltage‐sensing domains of the channel proteins and their conformations,^[^ ^137^ ^]^ the three‐step decrease gating is most likely associated with the stepwise conformational changes of the channels for the one‐way inbound DNA packaging process, while the three‐step gating of the current increase possibly reflects the stepwise conformational changes for the one‐way outbound ejection process.^[^ ^40^ ^]^ The high voltages used in these studies that go above the physiological membrane voltages (typically ranging from −40 to −80 mV) may generate artificial conformational changes in the portal proteins. Further investigations are required to exclude this possibility. Although the meaning of gating in the context of viral genome packaging and invasion is not fully understood, it can be assumed that translocation of the viral genome into the procapsid encounters the internal pressure of the packaged DNA that increases the probability of DNA contacts with the C‐terminal flexible domain. Such contacts induce conformation changes with the stepwise gating reduction to avoid dsDNA leakage from the capsid. It is also possible for the connector‐DNA contacts to prepare for ejection during the infection process. The ejection force may mostly build up during DNA packaging.^[^ ^138^ ^]^

### 4.4 Force generation of the hexameric ATPase involves electrostatic potentials

The binding of ATP to the gp16 ATPase is most likely mediated by electrostatic force.^[^ ^139^ ^]^ The electrostatic force from ATP binding is likely to affect the ATPase conformation.^[^ ^140^ ^]^ The binding of ATP increases the affinity of the ATPase for DNA,^[^ ^3^, ^42^ ^]^ as revealed by studying the electrostatic potential of the hexameric RecA‐ATP complex. RecA in this complex displays a high positive potential along the internal region in which the negatively charged ssDNA is bound.^[^ ^141^ ^]^ This suggests that the ATP‐bound conformation of the ATPase has a higher affinity for DNA than the unbound ATPase as a result of the increased positive charge on the DNA binding surface of the ATPase. One possible mechanism for the ATP‐binding increased affinity of the ATPase for DNA is dielectric allostery.^[^ ^142^ ^]^ Particularly, the dielectric allostery model was derived from molecular dynamics simulations of myosin in response to ATP binding, which is essential for force generation. Upon ATP binding to a motor protein of allostery such as myosin, the net negative charge of ATP induces such an extensive anisotropic dielectric response in the protein that the electrostatic potential in a distant region of that protein is altered. This alteration includes the increase of the positive charge of the actin‐binding loop and retraction of the loop.^[^ ^142^ ^]^ Accordingly, the ATP binding to the DNA motor proteins increases the affinity of the ATPase for DNA plausibly through the dielectric allostery. The binding of the gp16 ATPase to DNA is possibly regulated allosterically by ATP binding, the allosteric regulation that has been found in the DNA motor systems of the helicase superfamily II.^[^ ^143^, ^144^ ^]^ Besides, the DNA binding to the ATPase facilitates ATP hydrolysis by the ATPase,^[^ ^42^, ^145^ ^]^ plausibly by another allosteric mechanism as observed in the ATPase activity of *Escherichia coli* Rep helicase.^[^ ^146^, ^147^ ^]^ After ATP hydrolysis, the ATPase becomes unbound, and the conformation changes back to the initial state, which has a low affinity for DNA.^[^ ^3^, ^42^ ^]^ This low affinity conformation ATPase could generate a negatively charged binding surface that repels the negatively charged DNA substrate.^[^ ^148^ ^]^ The ATPase loses its affinity to dsDNA, and dsDNA departs for the next subunit of the ATPase. Taken together, the motor ATPase may be subjected to the allosteric regulations by the binding of the two substrates, ATP and DNA. ATP binding increases ATPase affinity for DNA by adding positive charges to the DNA binding surface of the ATPase via dielectric allostery. The bound DNA, in turn, facilitates ATP hydrolysis that reduces ATPase affinity for DNA. Whether the dielectric allostery is involved in dsDNA translocation by the DNA motors remains to be determined.

### 4.5 Translocation of dsDNA through nanopores

The phage connector can function as a nanopore that translocates DNA. Initially, it was proposed that the nanopore acted similarly to a nut and thus dsDNA as a bolt, using a rotational force to drive dsDNA into the procapsid.^[^ ^149^, ^150^ ^]^ However, several key experiments discovered that the rotation mechanism was unlikely for nanopores.

For the rotational mechanism to be true, either the nanopore or DNA would have to rotate. Bauman et al. tested this by creating several different constructs of a protein found in the nanopore. These constructs had regions that were tethered to the head of the bacteriophage so that the nanopore could not spin. However, even with the nanopore immobilized, the bacteriophage was still able to package DNA.^[^ ^151^ ^]^ The authors acknowledged that in these studies, the force with which DNA travels through the pore might be able to sever the engineered crosslinks, leaving room for debate on whether the rotation mechanism was viable. An additional study aimed to further investigate the mechanism of DNA transport. Hugel et al. used single‐molecule fluorescence spectroscopy to observe whether the nanopore rotated with respect to the viral prohead.^[^ ^152^ ^]^ In this work, several fluorescent dye molecules were attached to the nanopore. The prohead was immobilized on a glass coverslip. Magnetic beads were attached to dsDNA molecules, and magnets were placed on the objective of a fluorescent microscope to stretch the DNA polymer toward the objective. Even with the magnetic bead on dsDNA and the dye molecules on the nanopore, the nanopore was able to package DNA under the precaution that the fluorescent label on the nanopore could not move substantially on its own. Particularly, the excitation source was rotated in two orthogonal directions for polarization measurements. Changes in excitation polarization were considered for the dye observation. Upon incubation with ATP‐γS, a nonhydrolyzable ATP analog, the nanopore failed to transport DNA. During this incubation, the signal intensity of the dye remained unchanged based on variations in excitation polarization. This observation indicated that the dye molecule could not move by itself. In the presence of ATP, the nanopore was able to transport DNA as reflected by the fluorescent signal coming from the magnetic bead. However, the emission intensities of the dye molecules on the nanopore did not change, demonstrating that the nanopore remained still.^[^ ^152^ ^]^

Moreover, if the nanopore and the dsDNA were acting as a nut and bolt, the diameter of the nanopore would fit snugly around the dsDNA so that the nanopore and dsDNA would be “threaded” in the same chirality. Yet the experimental results with nanopores did not support this thought. Rotational biomotors display channels smaller than 2 nm, similar to the diameter of dsDNA. However, crystal structures of the phi29 channel diameter reveal 3.6 nm, larger than that of dsDNA. Furthermore, the channel displays a left‐handed chirality, opposite to the right‐handed chirality of dsDNA.^[^ ^5^, ^120^, ^153^ ^]^ Taken together, the data indicate that the translocation of dsDNA through nanopores most likely operates in the revolving mechanism.

## 5 CONCLUSION AND PERSPECTIVES

The architectural and functional similarities are unveiled in many eukaryotic and bacterial motors. Both types of motors have asymmetrical hexameric rings of ATPases with the internal diameters greater than the external diameter of dsDNA. The transacting arginine fingers are required for sequential DNA translocation through the revolving motion. In fact, the arginine finger acts as the starter of the motor to translocate dsDNA,^[^ ^43^ ^]^ other than the traditional belief that ATP is the starter to initiate a power stroke. Evidently, these nanomotors appear to operate in the revolving mechanism as exemplified by the phi29 motor to accomplish intricate transportation of the lengthy dsDNA genome. A summary of the biomotors discussed herein is presented in Table 1.

There is still much work to be done to further unravel the mechanisms of these biomotors and to harness them for biomedical applications. As further insight is gained into the molecular architecture and mechanisms of how these motors work, their properties can be fine‐tuned for specific uses. The phi29 biomotor is especially appealing since its components can be easily assembled in vitro.^[^ ^154^ ^]^ For example, the phi29 connector channel has been used for chemical sensing,^[^ ^155^ ^]^ identifying single amino acid differences in peptides,^[^ ^156^ ^]^ and nucleotide sequencing.^[^ ^157^ ^]^ Recently, an array of phi29 connector protein channels was inserted into a polymer membrane. This has the potential to be used for high‐throughput chemical sensing and peptide sequencing.^[^ ^158^ ^]^ Additionally, the discovery of the structural pRNA and its ability to form a variety of structures sparked the field of RNA nanotechnology. The pRNA has been used as building blocks for drug delivery vehicles^[^ ^159^, ^160^ ^]^ and as scaffoldings that act as “molecular circuit boards” to organize and regulate proteins.^[^ ^161^, ^162^ ^]^ The role of the arginine finger in the phi29 biomotor is becoming more defined, and information on how the arginine finger controls traffic through the phi29 channel yield valuable information on how to control the motion of biomotors.^[^ ^41^, ^76^ ^]^ The field of nanotechnology is advancing at a rapid pace as lessons are learned from nature's biomotors. Overall, the similarities and differences shown in the biomotors presented herein can lay the groundwork for the rational design of future nanomachines.

## CONFLICT OF INTEREST

Peixuan Guo is the consultant and licenser of Oxford Nanopore Technologies, and the cofounder and member of the Board of Directors of ExonanoRNA, LLC.
